# Variation of the serum *N*‐glycosylation during the pregnancy of a MPI‐CDG patient

**DOI:** 10.1002/jmd2.12247

**Published:** 2021-09-17

**Authors:** Elodie Lebredonchel, Sandrine Duvet, Claire Douillard, François Foulquier, André Klein

**Affiliations:** ^1^ UGSF—Unité de Glycobiologie Structurale et Fonctionnelle, UMR 8576 Université de Lille, CNRS Lille France; ^2^ Pôle Biologie Pathologie Génétique, Institut de Biochimie et de Biologie Moléculaire, UAM de Glycopathologies Université de Lille, CHU Lille Lille France; ^3^ Centre de Référence des Maladies Héréditaires du Métabolisme, Hôpital Huriez CHU de Lille, Département d'Endocrinologie et Métabolisme Lille France

**Keywords:** CDG, glycosylation, mannose, MPI, pregnancy, transferrin

## Abstract

For the first time the glycosylation of a patient with a MPI‐CDG during pregnancy is monitored. MPI‐CDG, is characterised by a deficiency in mannose‐6‐phosphate isomerase (MPI) leading to a reduced pool of glycosylation precursors, impairing the biosynthesis of *N*‐glycans leading to *N*‐glycosylation defects. The abnormal *N*‐glycosylation profile with an elevation of asialotransferrin and disialotransferrin, typical of CDG type I, is assessable by transferrin isoelectrofocusing. Oral D‐mannose supplementation for MPI‐CDG patients has been widely used and improves clinical manifestations. The glycosylation of a MPI‐CDG patient during pregnancy without mannose supplementation was studied using carbohydrate deficient transferrin (CDT) assay, transferrin isoelectrofocusing (IEF) and mass spectrometry of total serum *N*‐glycans. A general improvement of the glycosylation profile of the patient due to a better transfer of the glycan precursors as well as an increase of the triantennary glycans (and sialylation) was observed. In conclusion, in the absence of mannose supplementation, the previously observed glycosylation abnormality of the MPI‐CDG patient was corrected. The molecular mechanism underlying this *N*‐glycosylation rescue during MPI‐CDG pregnancy further needs to be investigated.

**Synopsis**

We monitored the glycosylation of a MPI‐CDG patient during the pregnancy without mannose therapy through the study of glycosylation and glycan structures. Interestingly we observed a correction of the glycosylation defect along the pregnancy.

## 1 INTRODUCTION

Congenital disorders of glycosylation (CDG) are a rapidly expanding family of rare inborn errors of metabolism. The first cases were reported 40 years ago^1^ and today more than 130 different CDG have been reported.^2^ MPI‐CDG, formerly named CDG‐Ib, is characterised by a deficiency in mannose‐6‐phosphate isomerase (MPI) due to autosomal recessive mutations in the *MPI* gene coding for the phosphomannose isomerase.^3^ MPI catalyses the reversible (inter)conversion of fructose‐6‐phosphate into mannose‐6‐phosphate (Man6‐P). The MPI defect results in a reduced pool of Man6P when mannose is not sufficient. As a consequence, the biosynthesis of GDP‐mannose and the lipid‐linked oligosaccharide (LLO) precursors pools, necessary for the biosynthesis of *N*‐glycans, are reduced leading to a defect of *N*‐glycosylation.^4^ Using isoelectrofocusing (IEF), an abnormal glycosylation profile of serotransferrin is detected for MPI‐CDG patients with an elevation of asialotransferrin and disialotransferrin, typical of CDG type I.^3^ The clinical manifestations are broad,^5^, ^6^, ^7^, ^8^ and are improved by oral D‐mannose supplementation.^9^, ^10^ Symptoms usually improve with age. Normal pregnancies have already been observed in MPI‐CDG patients.^11^, ^12^ Variations of glycosylation during pregnancy in healthy women have been described and might impact the immunity and the anti‐inflammatory response.^13^, ^14^, ^15^ In the present article, we have evaluated for the first time the *N*‐glycosylation variations during the pregnancy without mannose supplementation of a MPI‐CDG patient using carbohydrate deficient transferrin (CDT) assay, transferrin isoelectrofocusing (IEF) and mass spectrometry of total serum *N*‐glycans. This study aims to improve the pregnancy follow‐up of patient with glycosylation disorders and further questions the general mechanisms of glycosylation improvement during pregnancy.

## 2 MATERIALS

### 2.1 Samples

In order to check heparinemia, serum samples of a MPI‐CDG patient were collected every other week from the 7th to the 37th week of pregnancy for a total of 16 samples. Additionally, one sample before the pregnancy was collected as a control. This was made in agreement with the ethical policy of the institution.

## 3 METHODS

### 3.1 IEF transferrin

Transferrin isoelectrofocusing was performed on agarose on a Phast System (Amershan Biosciences) as previously described.^16^

### 3.2 Immunological determination of CDT

CDT levels were measured as a percentage of total transferrin (%CDT) using an automated nephelometric technique on a BN Prospec analyser and N Latex CDT immunoassay reagents (Siemens Healthcare, Marburg, Germany).

### 3.3 Release, permethylation and mass spectrometry analysis of *N*‐glycans

*N*‐glycans were prepared and released by PNGase F from an aliquot of 20 μL of serum as previously described.^17^ Released glycans were then permethylated, extracted and purified on a Sep‐Pak column as previously described.^18^ Permethylated glycans were solubilised in H_2_O/CH_3_OH (1:1, v/v) and spotted with 2,5 dihydroxybenzoic acid matrix solution (10 mg/mL dissolved H_2_O/CH_3_OH (1:1, v/v) on MALDI plate. MALDI‐TOF‐MS was acquired on 4800 MALDI TOF/TOF analyser (Applied Biosystems, Framingham, Massachusetts). For each sample, 5000 laser shots were accumulated.

## 4 CASE REPORT

First symptoms of the patient occurred at the age of 4 years and included early postprandial hypoglycemia due to non‐focal hyperinsulinism. The patient was treated by diazoxide until the age of 6, when MPI‐CDG diagnosis was established by transferrin isoelectrofocusing. At this time, there was a mild hepatomegaly with normal liver enzymes levels. D‐mannose treatment was started at 0.17 g/kg 4 times/day. Follow‐up in adolescence was very difficult with prolonged periods of non‐compliance. First episodes of deep venous thrombosis of lower limbs started at the age of 18. At 23, she presented a miscarriage and a deep venous thrombosis of lower limb. A new pregnancy was diagnosed at 7 week of amenorrhea and she was treated by apixaban and D‐mannose. After multidisciplinary discussion, D‐mannose was withdrawn to avoid a teratogenic risk.^19^ Apixaban was switched to a subcutaneous fractionated heparin (170 U/kg) throughout the pregnancy.

Pregnancy was successful despite a weight gain of 20 kg, pyelonephritis and a third trimester proteinuria (1.5 g/day) without preeclampsia. There was no deep venous thrombosis. She delivered by caesarean section for cervical dystocia at 41 weeks of amenorrhea. The child was a male of 3540 g, APGAR 10/10. The child was breastfed for several weeks (exact duration unknown). He is now 2 years old and has a normal growth.

Liver enzymes levels were normal during and after pregnancy and fibroscan did not show any fibrosis. Three months after delivering, proteinuria has disappeared and subcutaneous fractionated heparin was switched to apixaban. It is important to note that the patient had no chronic alcohol consumption before pregnancy.

## 5 RESULTS

### 5.1 Variation of the level of carbohydrate deficient transferrin during the pregnancy of the MPI‐CDG patient

Concentrations of transferrin and carbohydrate deficient transferrin (CDT) were assessed during the pregnancy of the MPI‐CDG patient. Human serotransferrin contains two *N*‐glycosylation sites, located on the asparagine residues Asn413 and Asn611.^20^ While IEF analysis is based on the number of negatively charged sialic acid residues terminating the two potential glycans attached to the transferrin backbone, CDT analysis corresponds to the amount of transferrin having lost one or two complete *N*‐glycan chain(s). The ratio of transferrin lacking complete *N*‐glycan chain(s) is expressed as a percentage (%CDT) and is assessed using an immunonephelometric technique with antibodies directed against the apotransferrin *N*‐glycosylation sites. The recognition of the peptide of the transferrin devoid of glycan allows to differentiate CDG type I from CDG type II.

**FIGURE 1:**
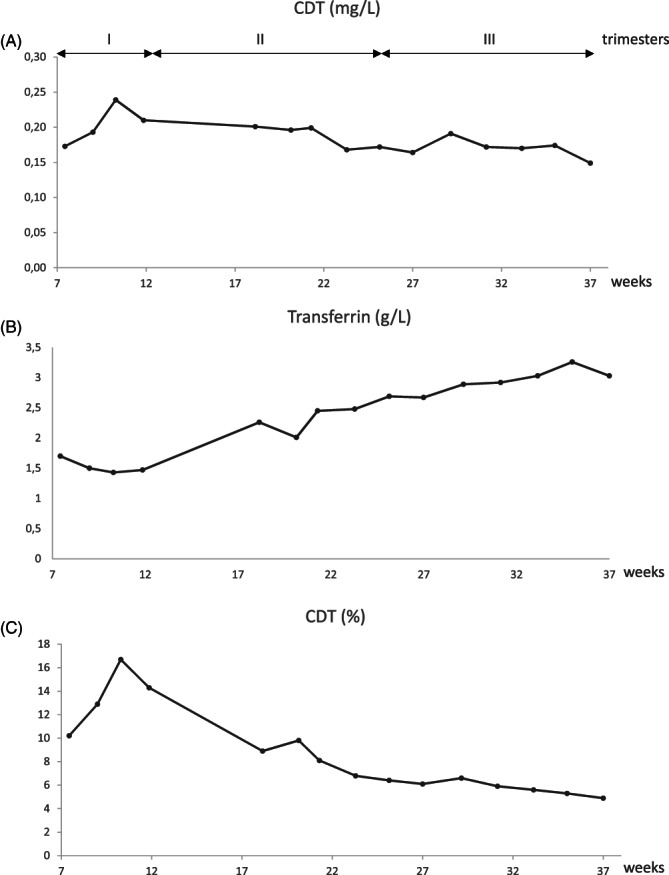
Evolution of the level of carbohydrate deficient transferrin and transferrin concentrations of the MPI‐CDG patient during pregnancy. Serum concentrations of CDT in mg/L (A), transferrin in g/L (B) and ratio CDT/transferrin in percentage (C)

During the first trimester of pregnancy, the amount of CDT increased (0.17–0.24 mg/L; Figure 1A) while the transferrin concentration decreased (1.7–1.47 g/L; Figure 1B), as a result the %CDT increased (10%–16.7%; Figure 1C). During the second and the third trimesters the CDT remained relatively stable (0.15–0.20 mg/L; Figure 1A) while the transferrin concentration continued to rise (1.47–3.03 g/L; Figure 1B) and consequently the %CDT decreased (12% to 4.9%; Figure 1C).

Overall there is a twofold increase of the transferrin concentration in the serum during the pregnancy of the MPI‐CDG patient, while the concentration of CDT remained relatively constant (Figure 1A,B); consequently, the ratio of %CDT decreased (Figure 1C). This indicates that during the pregnancy, the quantity of precursor tetradecasaccharide transferred on the apotransferrin by the oligosaccharyltransferase in the endoplasmic reticulum has doubled.

CDT has been assessed a year after the pregnancy of the patient. Interestingly, the results showed an increase of the CDT (0.38 mg/L) with a stable transferrin (1.75 g/L) resulting in high percentage of CDT N Latex (38%). This indicates that the glycosylation improvement observed in the patient during the pregnancy following %CDT was reversible without mannose therapy.

### 5.2 Variations in the transferrin isoforms distribution during the pregnancy of the MPI‐CDG patient

**FIGURE 2:**
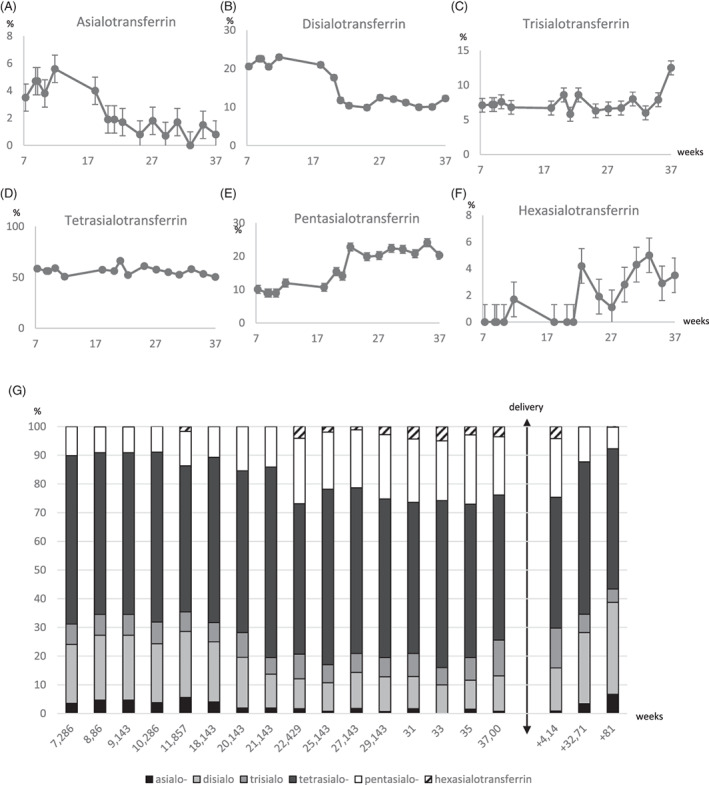
Changes in the transferrin glycoforms distribution of the MPI‐CDG patient during pregnancy. The distribution of transferrin subfractions expressed as a percentage in the sera of a MPI‐CDG patient during pregnancy. Proportion of asioalotransferrin (A), disialotransferrin (B), trisialotransferrin (C), tetrasialotransferrin (D), pentasialotransferrin (E), and hexasialotransferrin glycoforms (F). Representation of the % evolution of the transferrin glycoforms along the pregnancy (G). Asialotransferrin, disialotransferrin, trisialotransferrin, tetrasialotransferrin, pentasialotransferrin and hexasialotransferrin are, respectively, depicted by black, light grey, middle grey, dark grey, white and hatched bars

During the first trimester of pregnancy, the MPI‐CDG patient IEF profile presented very little modifications with the exception of an increase of the asialotransferrin relative amount (3.5%–5.6%; Figure 2A). During the second and the third trimesters of pregnancy, the relative amount of asialotransferrin (5.6%–0%) and disialotransferrin (22.6%–12.5%; Figure 2A,B) decreased concomitantly to an observed increase in the amount of the pentasialotransferrin (12%–24.1%) and hexasialotransferrin (+3.5%; Figure 2E,F) while the relative amount of trisialotransferrin (6.3%–12.5%) and tetrasialotransferrin (50.9%–66.4%) remained stable (Figure 2C,D). The evolution along pregnancy of the % of the transferrin glycoforms is described in Figure 2G and Figure S1.

The decrease of asialotransferrin and disialotransferrin confirms the observation made with the immunonephelometric technique (Figure 1).

Post‐partum transferrin IEF has been performed. The right side of the double arrow of Figure 2G shows the repartition of transferrin glycoforms a day, a month and a year after delivery. An increase of asialotransferrins (0.9%–6.7%), disialotransferrin (15%–32%) and a decrease of trisialotransferrin (13.9%–4.7%), pentasialotransferrin (20.4%–7.5%) and hexasialotransferrin (4.3%–0.3%) is observed. This indicates that the glycosylation improvement of the patient during the pregnancy assessed by transferrin IEF was transitory without mannose supplementation.

In an attempt to explain the increase of pentasialotransferrin and hexasialotransferrin forms, we analysed the nature and the distribution of the glycans present in the total serum *N*‐glycome by mass spectrometry.

### 5.3 Variations of the total serum *N*‐glycome

**FIGURE 3:**
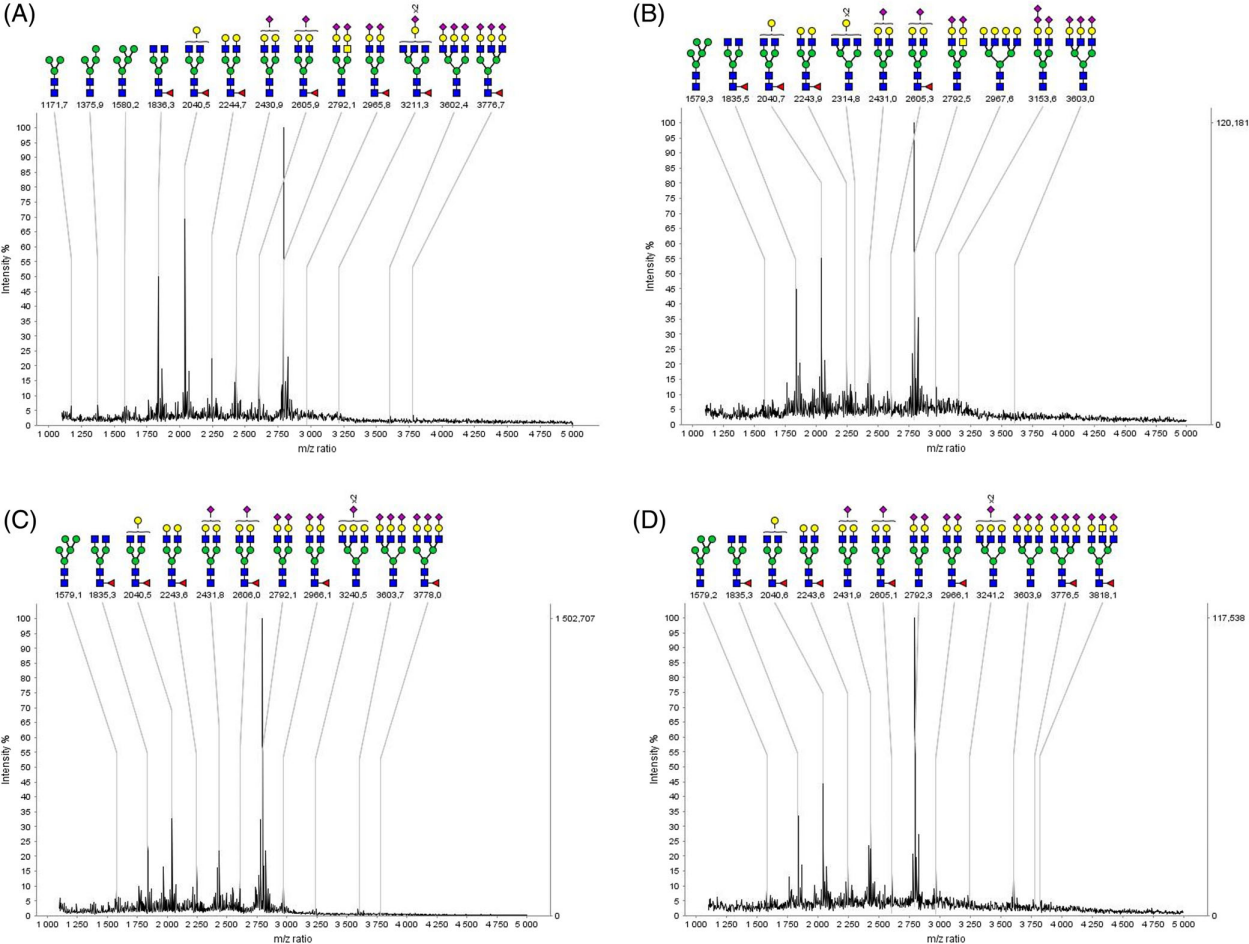
Serum *N*‐glycome evolution of the MPI‐CDG patient pregnancy. MALDI‐TOF analysis of permethylated serum *N*‐glycans released after PNGase‐F treatment, of a MPI‐CDG patient before pregnancy (A), during the first trimester (B), the second trimester (C) and the third trimester of pregnancy (D). Symbols: GlcNAc, blue square; Man, green circle; Gal, yellow circle; Neu5Ac, magenta diamond; Fucose, red triangle

Total serum *N*‐glycome was analysed by mass spectrometry (Figure 3). The glycosylation profile of the first trimester was similar to the control serum before pregnancy (Figure 3A,B). In the spectrum of the second trimester (Figure 3C) a relative increase of the triantennary structure present at m/z 3603 can be observed and confirmed in the profile of the serum of the third trimester (Figure 3D) showing an increase of the triantennary structures at m/z 3603 and 3777 compared to the second trimester. Overall, a higher proportion of the triantennary and the fucosylated triantennary *N*‐glycans between the first and the third trimester is observed, likely explaining the observed changes in transferrin IEF.

## 6 DISCUSSION

In the present study, we evaluated for the first time the *N*‐glycosylation variations during the pregnancy without mannose supplementation of a MPI‐CDG patient using three different techniques. A clear restoration of the glycosylation defect is observed during pregnancy and also an increase of the *N*‐glycan branching as observed in pregnant healthy women.

MPI‐CDG is characterised by the absence of neurological symptoms and is sometimes fortuitously diagnosed. A healthy women with one child has even been diagnosed during a routine company health checkup.^12^ Mannose supplementation is the recommended treatment for MPI‐CDG patients in Europe and the United States.^21^ However, excessive mannose administration in MPI‐CDG patients can cause accumulation of Man6‐P^22^ consequently leading to teratogenesis as observed in bees for the first time, in the so‐called '*honeybee syndrome*'.^23^, ^24^ The teratogenicity of mannose substitution has been evaluated in various species. In normal pregnant rat mothers infused with D‐mannose, dysmorphic changes were seen in embryos.^25^ In *PMI* KO mice, mannose substitution increased embryonic death.^19^, ^26^ Even for hypomorphic mice with a *PMI* residual activity, mannose led to blindness.^27^ In MPI‐CDG zebrafish, the addition of mannose in water restored glycosylation and reduced embryonic death.^28^ In humans, for more than 20 years, oral mannose administration has proven a clinical and biochemical improvement without major side effects.^3^ No foetal death has been reported so far, however, additional trials have to be conducted to assess the innocuousness of mannose therapy during pregnancy in humans. Moreover, a case of pregnancy in an undiagnosed MPI‐CDG patient without mannose supplementation has been previously reported.^12^ Therefore, mannose therapy has been interrupted for the MPI‐CDG patient.^11^

As the mannose supplementation was interrupted, an increase of hyposialylated transferrin was expected for the patient. This was observed during the first trimester of pregnancy. However, our results showed an increase in *N*‐glycan transfer as illustrated by the decrease of CDT during the second and third trimesters (Figure 1).

It is worth to know that during pregnancy, there is a raise in glycoprotein biosynthesis, the concentration of serotransferrin doubles in response to the increased demand of the fetoplacental unit and maternal bone marrow for iron transport.^29^, ^30^, ^31^ A slight increase of the percentage of CDT of approximately 0.5% has been described in healthy pregnant women.^32^ For CDG type I patients CDT ratios between 8% and 50% have been reported.^33^ In our patient, despite the phosphomannose isomerase deficiency, the concomitant increase of transferrin concentration and the stagnation of CDT during the pregnancy indicate a spontaneous suppression of the unoccupied glycosylation site(s). This can either be explained by a more efficient transfer of LLO precursors onto proteins by the oligosaccharyltransferase and/or the increase of the precursors pool required to form the lipid‐linked oligosaccharide (LLO), such as dolichol and GDP‐mannose, and/or an increase of the enzymes activities.

During pregnancy, the elevation of the serotransferrin concentration is associated with modifications of the *N*‐glycan microheterogeneity of transferrin.^34^, ^35^ In physiological conditions, the disialotransferrin ratio is around 2% and remains stable during pregnancy; the trisialotransferrin and tetrasialotransferrin tend to decrease, and the pentasialotransferrin and hexasialotransferrin rise sharply; asialotransferrin and monosialotransferrin are mostly absent and not visible on IEF.^29^, ^36^, ^37^ The study of the transferrin glycoforms of the MPI‐CDG patient during the pregnancy interestingly showed a similar evolution of the transferrin glycoforms with a strong decrease of the asialotransferrin and disialotransferrin concomitant to the increase of the pentatransferrin and hexasialotransferrin explained by an elevation of the triantennary glycans, similar to healthy women pregnancy.

Changes are thought to be influenced by hormones, such as estrogens and progestogens, on transferrin isoform distribution,^29^ but no evidence of a hormonal effect on the branching enzymes (*N*‐acetylglucosaminyltransferases IV and V) has been reported so far. Moreover, the increase of intracellular UDP‐GlcNAc has been shown to induce the increase of the number of antennae of the *N*‐glycans.^38^ Furthermore, in pregnancy there is an increased galactosylation of immunoglobulins in patients suffering from rheumatoid arthritis^15^, ^35^ leading to the remission of the clinical symptoms. All these glycosylation modifications, (a) the increase of the number of glycan antennae, (b) the increased galactosylation, and (c) the increased availability of the LLO precursors, might indicate a general mechanism of improved glycosylation linked to an increased metabolic flux during pregnancy and explain the normal pregnancy of the untreated MPI‐CDG patient. The molecular mechanism underlying this *N*‐glycosylation rescue during MPI‐CDG pregnancy further needs to be investigated.

**Abbreviations**
CDG: congenital disorders of glycosylation
CDT: carbohydrate deficient transferrin
GDP: guanosine diphosphate
IEF: isoelectrofocusing
LLO: lipid‐linked oligosaccharide
Man6‐P: mannose‐6‐phosphate
MPI: mannose phosphate isomerase

## CONFLICT OF INTEREST

E. Lebredonchel, S. Duvet, C. Douillard, F. Foulquier and A. Klein declare they have no conflict of interest.

## ETHICS STATEMENT

All procedures followed were in accordance with the ethical standards of the responsible committee on human experimentation (institutional and national) and with the Helsinki Declaration of 1975, as revised in 2000. Informed consent was obtained from all patients for being included in the study.

## Supporting information


**Figure S1** Observed changes in the distribution of transferrin glycoforms via transferrin IEF in the MPI‐CDG patient during pregnancy and in post‐partum numbers 0, 2, 3, 4, 5 and 6 indicate the migration position of the asialotransferrin, disialotransferrin, trisialotransferrin, tetrasialotransferrin, pentasialotransferrin and hexasialotransferrin forms, respectively.

## References

[1] Jaeken J , Vanderschueren‐Lodeweyckx M , Casaer P , et al. Familial psychomotor retardation with markedly fluctuating serum prolactin, FSH and GH levels, partial TBG‐deficiency, increased serum arylsulphatase a and increased CSF protein: a new syndrome?: 90. Pediatr Res. 1980;14:179. 10.1203/00006450-198002000-00117

[2] Jaeken J . Congenital disorders of glycosylation: a multi‐genetic disease family with multiple subcellular locations. J Mother Child. 2020;24:14‐20. 10.34763/jmotherandchild.20202402si.2005.000004 33554500 PMC8518092

[3] Niehues R , Hasilik M , Alton G , et al. Carbohydrate‐deficient glycoprotein syndrome type Ib, phosphomannose isomerase deficiency and mannose therapy. J Clin Invest. 1998;101:1414‐1420. 10.1172/JCI2350 9525984 PMC508719

[4] Ichikawa M , Scott DA , Losfeld M‐E , Freeze HH . The metabolic origins of mannose in glycoproteins. J Biol Chem. 2014;289:6751‐6761. 10.1074/jbc.M113.544064 24407290 PMC3945336

[5] Damen G , de Klerk H , Huijmans J , et al. Gastrointestinal and other clinical manifestations in 17 children with congenital disorders of glycosylation type Ia, Ib, and Ic. J Pediatr Gastroenterol Nutr. 2004;38:282‐287. 15076627 10.1097/00005176-200403000-00010

[6] de Lonlay P , Cuer M , Vuillaumier‐Barrot S , et al. Hyperinsulinemic hypoglycemia as a presenting sign in phosphomannose isomerase deficiency: a new manifestation of carbohydrate‐deficient glycoprotein syndrome treatable with mannose. J Pediatr. 1999;135:379‐383. 10.1016/S0022-3476(99)70139-3 10484808

[7] Pedersen PS , Tygstrup I . Congenital hepatic fibrosis combined with protein‐losing enteropathy and recurrent thrombosis. Acta Paediatr Scand. 1980;69:571‐574. 10.1111/j.1651-2227.1980.tb07136.x 7446108

[8] Pelletier VA , Galéano N , Brochu P , et al. Secretory diarrhea with protein‐losing enteropathy, enterocolitis cystica superficialis, intestinal lymphangiectasia, and congenital hepatic fibrosis: a new syndrome. J Pediatr. 1986;108:61‐65. 10.1016/S0022-3476(86)80769-7 3080572

[9] de Lonlay P , Seta N . The clinical spectrum of phosphomannose isomerase deficiency, with an evaluation of mannose treatment for CDG‐Ib. Biochim Biophys Acta. 2009;1792:841‐843. 10.1016/j.bbadis.2008.11.012 19101627

[10] Westphal V , Kjaergaard S , Davis JA , et al. Genetic and metabolic analysis of the first adult with congenital disorder of glycosylation type Ib: long‐term outcome and effects of mannose supplementation. Mol Genet Metab. 2001;73:77‐85. 10.1006/mgme.2001.3161 11350186

[11] Girard M , Douillard C , Debray D , et al. Long term outcome of MPI‐CDG patients on D‐mannose therapy. J Inherit Metab Dis. 2020;43:1360‐1369. 10.1002/jimd.12289 33098580

[12] Helander A , Jaeken J , Matthijs G , Eggertsen G . Asymptomatic phosphomannose isomerase deficiency (MPI‐CDG) initially mistaken for excessive alcohol consumption. Clin Chim Acta. 2014;431:15‐18. 10.1016/j.cca.2014.01.018 24508628

[13] Bondt A , Nicolardi S , Jansen BC , et al. Longitudinal monitoring of immunoglobulin a glycosylation during pregnancy by simultaneous MALDI‐FTICR‐MS analysis of N‐ and O‐glycopeptides. Sci Rep. 2016;6:27955. 10.1038/srep27955 27302155 PMC4908400

[14] Larsson A , Palm M , Hansson L‐O , et al. Reference values for alpha1‐acid glycoprotein, alpha1‐antitrypsin, albumin, haptoglobin, C‐reactive protein, IgA, IgG and IgM during pregnancy. Acta Obstet Gynecol Scand. 2008;87:1084‐1088. 10.1080/00016340802428146 18792844

[15] Reiding KR , Vreeker GCM , Bondt A , et al. Serum protein N‐glycosylation changes with rheumatoid arthritis disease activity during and after pregnancy. Front Med. 2018;4:241. 10.3389/fmed.2017.00241 PMC5766648 29359131

[16] Carchon H , Van Schaftingen E , Matthijs G , Jaeken J . Carbohydrate‐deficient glycoprotein syndrome type IA (phosphomannomutase‐deficiency). Biochim Biophys Acta. 1999;1455:155‐165. 10.1016/S0925-4439(99)00073-3 10571009

[17] Duvet S , Mouajjah D , Péanne R , et al. Use of endoglycosidase H as a diagnostic tool for MAN1B1‐CDG patients. Electrophoresis. 2018;39:3133‐3141. 10.1002/elps.201800020 29947113

[18] Faid V , Chirat F , Seta N , et al. A rapid mass spectrometric strategy for the characterization of N‐ and O‐glycan chains in the diagnosis of defects in glycan biosynthesis. Proteomics. 2007;7:1800‐1813. 10.1002/pmic.200600977 17520685

[19] Sharma V , Nayak J , DeRossi C , et al. Mannose supplements induce embryonic lethality and blindness in phosphomannose isomerase hypomorphic mice. FASEB J. 2014b;28:1854‐1869. 10.1096/fj.13-245514 24421398 PMC3963023

[20] MacGillivray RT , Mendez E , Shewale JG , et al. The primary structure of human serum transferrin: the structures of seven cyanogen bromide fragments and the assembly of the complete structure. J Biol Chem. 1983;258:3543‐3553. 6833213

[21] Brasil S , Pascoal C , Francisco R , et al. CDG therapies: from bench to bedside. Int J Mol Sci. 2018;19:1304. 10.3390/ijms19051304 PMC5983582 29702557

[22] de la Fuente M , Peñas PF , Sols A . Mechanism of mannose toxicity. Biochem Biophys Res Commun. 1986;140:51‐55. 10.1016/0006-291x(86)91056-9 3096320

[23] Freinkel N , Lewis NJ , Akazawa S , et al. The honeybee syndrome ‐ implications of the teratogenicity of mannose in rat‐embryo culture. N Engl J Med. 1984;310:223‐230. 10.1056/NEJM198401263100404 6690938

[24] Sols A , Cadenas E , Alvarado F . Enzymatic basis of mannose toxicity in honey bees. Science. 1960;131:297‐298. 10.1126/science.131.3396.297 13832710

[25] Buchanan T , Freinkel N , Lewis NJ , et al. Fuel‐mediated teratogenesis: use of D‐mannose to modify organogenesis in the rat embryo in vivo. J Clin Invest. 1985;75:1927‐1934. 10.1172/JCI111908 2409111 PMC425550

[26] DeRossi C , Bode L , Eklund EA , et al. Ablation of mouse phosphomannose isomerase (Mpi) causes mannose 6‐phosphate accumulation, toxicity, and embryonic lethality. J Biol Chem. 2006;281:5916‐5927. 10.1074/jbc.M511982200 16339137

[27] Sharma V , Ichikawa M , Freeze HH . Mannose metabolism: more than meets the eye. Biochem Biophys Res Commun. 2014a;453:220‐228. 10.1016/j.bbrc.2014.06.021 24931670 PMC4252654

[28] Chu J , Mir A , Gao N , et al. A zebrafish model of congenital disorders of glycosylation with phosphomannose isomerase deficiency reveals an early opportunity for corrective mannose supplementation. Dis Model Mech. 2013;6:95‐105. 10.1242/dmm.010116 22899857 PMC3529342

[29] de Jong G , van Noort WL , Feelders RA , et al. Adaptation of transferrin protein and glycan synthesis. Clin Chim Acta. 1992;212:27‐45. 10.1016/0009-8981(92)90135-d 1486679

[30] Fletcher J , Suter PE . The transport of iron by the human placenta. Clin Sci. 1969;36:209‐220. 5772100

[31] Laurell CB , Kullander S , Thorell J . Effect of administration of a combined estrogen‐progestin contraceptive on the level of individual plasma proteins. Scand J Clin Lab Invest. 1968;21:337‐343. 10.3109/00365516809077003 4178691

[32] Kenan N , Larsson A , Axelsson O , Helander A . Changes in transferrin glycosylation during pregnancy may lead to false‐positive carbohydrate‐deficient transferrin (CDT) results in testing for riskful alcohol consumption. Clin Chim Acta. 2011;412:129‐133. 10.1016/j.cca.2010.09.022 20869959

[33] Colomé C , Ferrer I , Artuch R , et al. Personal experience with the application of carbohydrate‐deficient transferrin (CDT) assays to the detection of congenital disorders of glycosylation. Clin Chem Lab Med. 2000;38:965‐969. 10.1515/CCLM.2000.143 11140630

[34] Léger D, Campion B, Decottignies JP, Montreuil J, Spik G. Physiological significance of the marked increased branching of the glycans of human serotransferrin during pregnancy. Biochem J. 1989;257(1):231‐8. 10.1042/bj2570231 2920013 PMC1135560

[35] de Jong G , van Eijk HG . Microheterogeneity of human serum transferrin: a biological phenomenon studied by isoelectric focusing in immobilized pH gradients. Electrophoresis. 1988;9:589‐598. 10.1002/elps.1150090921 3243256

[36] van Eijk HG , van Noort WL , de Jong G , Koster JF . Human serum sialo transferrins in diseases. Clin Chim Acta. 1987;165:141‐145. 10.1016/0009-8981(87)90157-4 3652443

[37] van Eijk HG , van Noort WL , Dubelaar ML , van der Heul C . The microheterogeneity of human transferrins in biological fluids. Clin Chim Acta. 1983;132:167‐171. 10.1016/0009-8981(83)90244-9 6616871

[38] Grigorian A , Lee S‐U , Tian W , et al. Control of T cell‐mediated autoimmunity by metabolite flux to N‐glycan biosynthesis. J Biol Chem. 2007;282:20027‐20035. 10.1074/jbc.M701890200 17488719

