# Supplementary figures and images for: Variation of the serum N‐glycosylation during the pregnancy of a MPI‐CDG patient
Source: JIMD Rep. 2021 Sep 17;62(1):22–9. doi: 10.1002/jmd2.12247 (PMC8574185; doi:10.1002/jmd2.12247)

Supp. fig. S-1

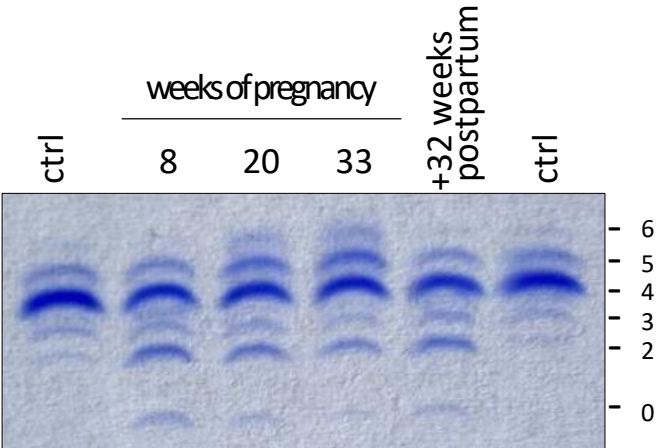

Supplement: Supplementary file 1 — Figure S1 Observed changes in the distribution of transferrin glycoforms via transferrin IEF in the MPI‐CDG patient during pregnancy and in post‐partum numbers 0, 2, 3, 4, 5 and 6 indicate the migration position of the asialotransferrin, disialotransferrin, trisialotransferrin, tetrasialotransferrin, pentasialotransferrin and hexasialotransferrin forms, respectively. [file JMD2-62-22-s001.pdf]
